# Royal jelly peptides: potential inhibitors of β-secretase in N2a/APP695swe cells

**DOI:** 10.1038/s41598-018-35801-w

**Published:** 2019-01-17

**Authors:** Xueqing Zhang, Yi Yu, Ping Sun, Zhen Fan, Wensheng Zhang, Chengqiang Feng

**Affiliations:** Beijing Key Laboratory of Protection and Utilization of Chinese Medicine, Beijing Normal University, Beijing, People’s Republic of China

## Abstract

Royal jelly (RJ) is a type of natural health product with a long history of use. Royal jelly peptides (RJPs) obtained from RJ have numerous bioactivities. To study the neuroprotective effect of RJPs, major royal jelly proteins were digested into crude RJPs and subsequently purified by RP-HPLC. Purified RJP fractions were evaluated in N2a/APP695swe cells. Our results indicated that purified royal jelly peptides (RJPs) (1–9 μg/mL) could inhibit external beta-amyloid 40 (Aβ_1-40_) and beta-amyloid 42 (Aβ_1-42_) production through the down-regulation of β-secretase (BACE1) in N2a/APP695 cells. The modulation of BACE1 may be related to histone acetylation modification. Our results demonstrated a neuroprotective function of RJPs, which indicates that RJPs may serve as potential β-secretase inhibitors in ameliorating Aβ-related pathology in Alzheimer’s Disease.

## Introduction

Royal jelly (RJ) is a well-known honeybee product secreted by the hypopharyngeal and mandibular glands of the worker honeybees and has been documented to have a wide-range of usages for promoting human health^1,2^. RJ has been recognized as having several pharmacological properties, including anti-hypercholesterolemic^3^, and antioxidant^4^ abilities among others^5,6^. The main dry matter of royal jelly consists of royal jelly proteins^7^. The dimorphism of honeybee development is known to depend not on genetic differences but on the ingestion of royal jelly. A 57-kDa protein in royal jelly has been found to play an important role in inducing the differentiation of honeybee larvae into queens^8^. Moreover, studies have shown that royal jelly peptides (RJPs) digested from royal jelly proteins have antimicrobial, immunomodulatory, antioxidative, and antihypertensive effects^9–12^. However, few studies have focused on the neuroprotective effect of RJPs on nerve cells.

Alzheimer’s Disease (AD) is one of the most common neurodegenerative diseases, which are characterized by loss of memory and recognition ability and movement dysfunction^13^. Its pathological features are extracellular senile plaques and intracellular neurofibrillary tangles^14^. There are two main hypotheses to explain the pathological mechanism of AD: the beta-amyloid peptide (Aβ) cascade hypothesis and the tau protein hypothesis^15,16^. Moreover, numerous studies have indicated that an abnormal metabolism of Aβ and its toxic aggregation can lead to the symptoms of AD^17^. β-secretase (BACE1) has been discovered to initiate the cleavage of amyloid precursor protein (APP) at the β-secretase site. Only after this cleavage does γ-secretase further cleave the BACE1-cleaved C-terminal APP fragment to release Aβ^18–20^. Thus, several chemicals have been found that can restrain the expression of BACE1 and its cleavage activity to reduce the accumulation of Aβ, which has been thought be useful for relieving AD^21,22^. N2a/APP695 cells (N2a cells stably transfected with the human APP gene) are widely used *in vitro* model of Aβ production by amyloidogenesis pathway^23^. These cells can produce more APP, which is subsequently cleaved into Aβ, similar to the AD pathology.

Recently, neuroepigenetics has provided evidence to indicate that epigenetic modifications play a significant role in AD^24^. In sporadic AD patients, AD-related genes such as APP and MAPT (Microtubule-Associated Protein Tau) show intense CpG methylation^25^. In addition, studies have suggested that AD-related genes, such as BACE1 and PS1, show increased histone H3 acetylation in their promoter region, which activates expression of these genes, in cell and animal models^26,27^. Thus, these studies provide a novel way to cure AD or prevent the process of AD. A recent study indicated that galangin, a natural flavonoid, can significantly lower Aβ levels through the inhibition of BACE1 by decreasing histone acetylation modification.

Although numerous studies have focused on the antioxidant, antimicrobial and immunomodulatory effect of RJ or RJPs, only a few studies have reported on the neuroprotective effects of RJPs. In this regard, this study mainly investigates the neuroprotective effect of RJPs digested from royal jelly proteins on nerve cells. First, water-soluble RJPs were digested by bee larva entero-enzymes (intestinal canal enzyme solution). Then, crude RJPs were fractioned into various components using high-performance liquid chromatography (RP-HPLC) methods. Furthermore, purified RJPs were investigated in N2a/APP695 cells to explore their effects on the metabolism of Aβ and the possible mechanism. This work provides new evidence that RJPs obtained from royal jelly have neuroprotective functions in some nerve cells and could be serve as novel natural BACE1 inhibitors, which may provide beneficial effects for AD patients.

## Results

### Preparation of crude RJPs from digested water-soluble royal jelly (WSRJ) proteins by intestinal enzymes

**Figure 1:**
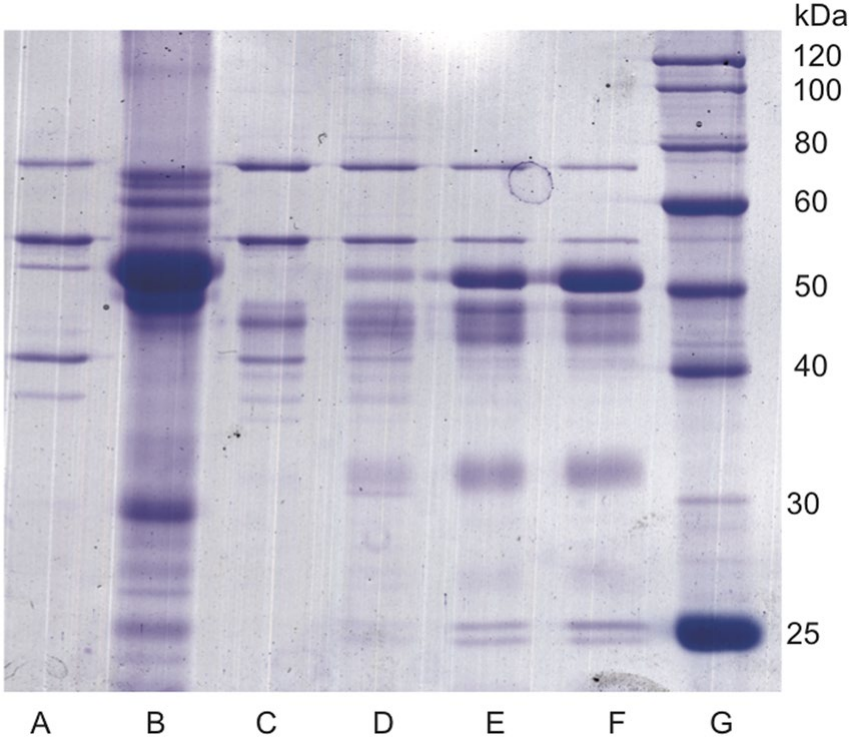
SDS-PAGE analysis of honey bee larva intestinal enzymes, major royal jelly proteins (MRJPs) and digested royal jelly proteins. Honey been larva intestinal enzymes (lane A) were obtained from honey bee larva, major royal jelly proteins (lane B) were obtained from soluble royal jelly. Mixture of digested royal jelly proteins and intestinal enzymes were shown as lane C, D, E and F. Digestion condition of lane D was selected for preparation of royal jelly peptides. Protein lander was shown as lane G.

Honey bee larva intestinal enzymes and WSRJ proteins were obtained as described (Fig. 1). SDS-PAGE results showed that MRJPs of WSRJ proteins were fully digested into crude RJPs by intestinal enzymes. Afterwards, crude RJPs were separated into three different constituents according to molecular weight(MW) via an ultra-filtration method, namely, MW < 1-kDa, 1-3-kDa and 3-5-kDa RJPs (named after the molecular weight).

### Purification of fraction 1 C^I^RJP by prep-RPLC, semi-prep-RPLC and RP-HPLC

**Figure 2:**
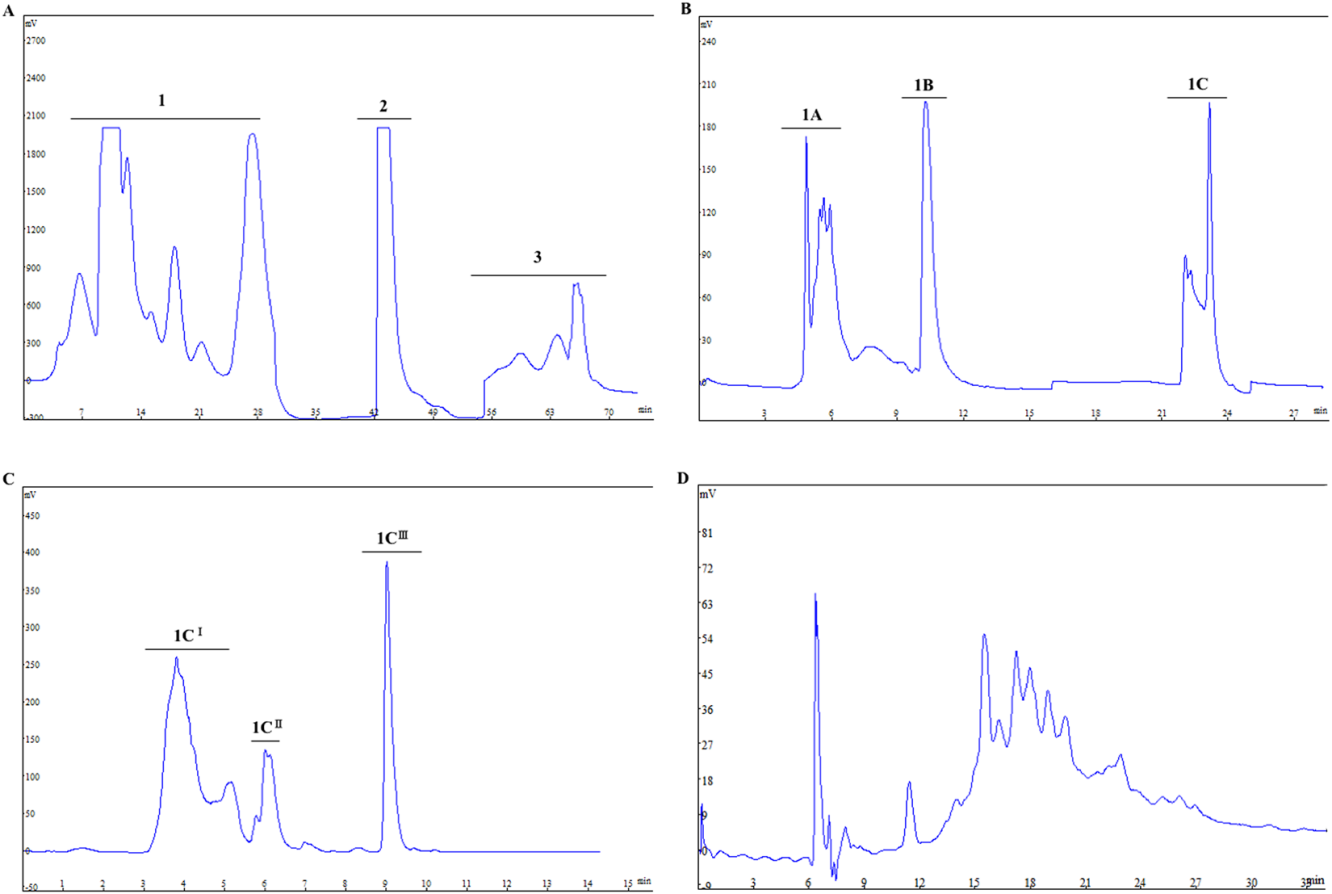
Separation of RJPs by prep-HPLC, semi-prep-HPLC and RP-HPLC. Representative HPLC chromatogram for initial separation of 1-3-kDa RJPs (**A**), purification of fraction 1 (**B**), separation of fraction 1 C (**C**) and analysis of fraction 1 C^I^ (**D**).

Crude RJPs were tested for their anti-apoptotic or antioxidant properties. Here, 1-3-kDa RJPs and 3-5-kDa RJPs were recognized as active components (see below). Then, 1-3-kDa RJPs were purified using the prep-RPLC and semi-prep-RPLC method. We obtained three fractions from the 1-3-kDa RJPs (Fig. 2A). These three components were evaluated by preliminarily bioactivity analysis such as a cell viability assay. Then, fraction 1 was chosen to be purified in the next step, and fraction 1 A, 1 B and 1 C were obtained (Fig. 2B). Fraction 1 C was separated after the evaluation of its bioactivity, and we acquired fraction 1 C^I^, 1 C^II^, 1 C^III^ (Fig. 2C). Finally, fraction 1 C^I^ was recognized as active peptides (see below). Even though we obtained purified RJPs from the original 1-3-kDa RJPs via different resolutions of RP-HPLC, 1 C^I^ still contained multiple types of peptides (Fig. 2D). Considering the limited amount 1 C^I^ RJPs, we did not perform further purification procedure. Finally, 1 C^I^ RJPs were resolved in deionized water to investigate their functions.

### Fraction 1 C^I^RJP inhibits extracellular Aβ1-40 and Aβ1-42 accumulation in N2a/APP695 cells

**Figure 3:**
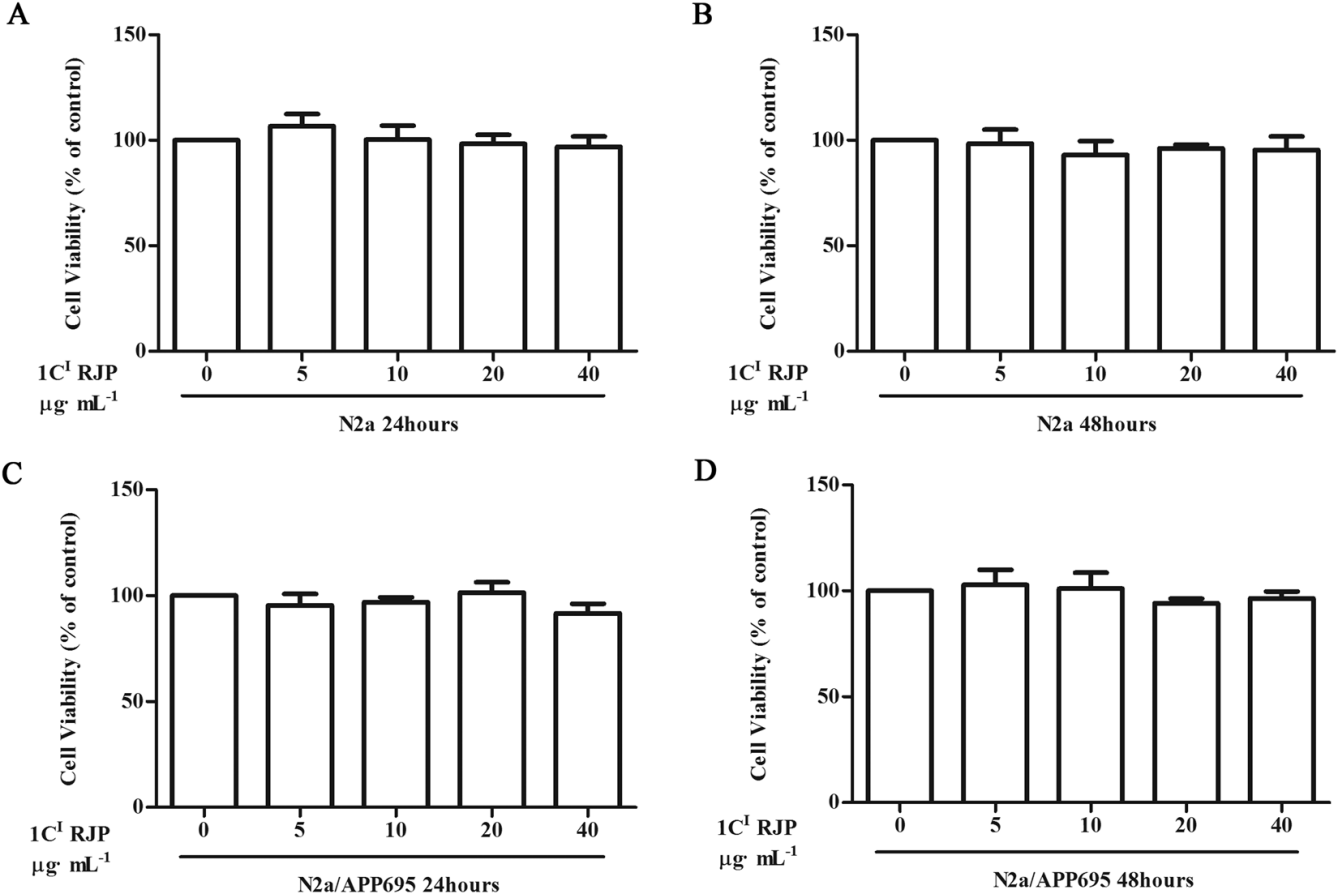
Fraction 1 C^I^RJPs is not toxic for N2a and N2a/APP695 cells. MTT assay was used to assess cell viability of N2a cells treated with different concentrations of fraction 1 C^I^ of RJPs (1 C^I^ RJP) for 24 hours (**A**) and 48 hours (**B**), and cell viability of N2a/APP695 cells treated with 1 C^I^ RJP for 24 hours (**C**) and 48 hours (**D**). Non-treated N2a cells were considered as control. Data are expressed as the mean ± SD of three independent experiments. Statistical analyses were performed by a one-way ANOVA test and the addition of Bonferroni multiple comparisons test.

**Figure 4:**
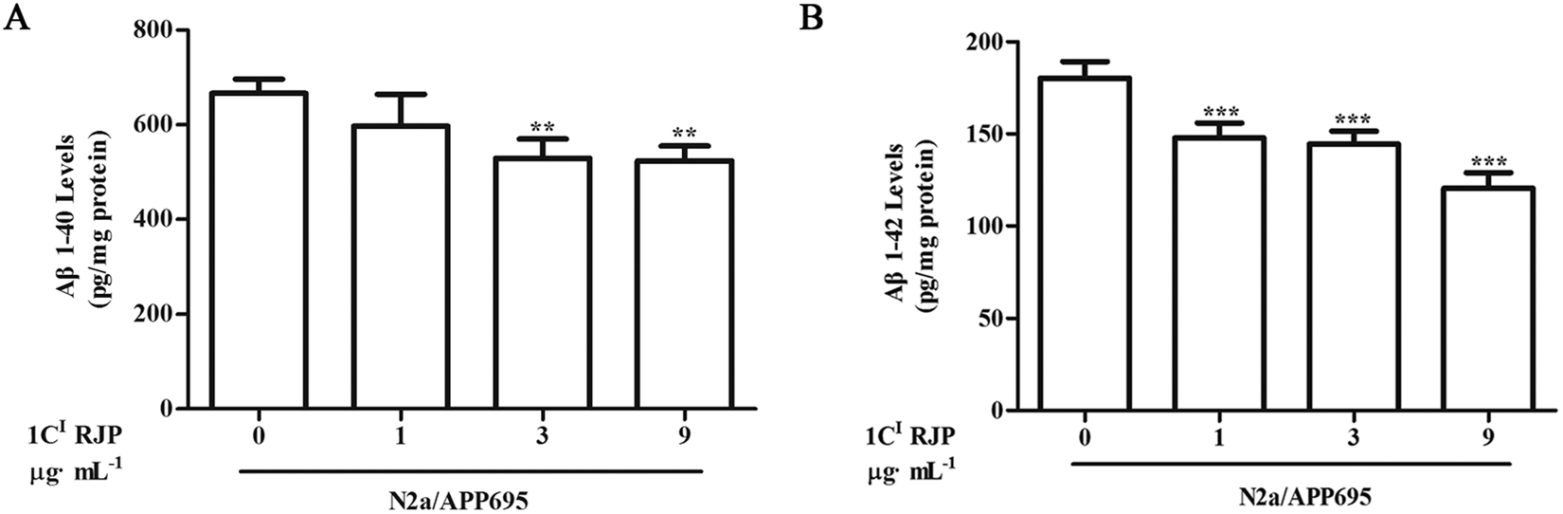
Fraction 1 CI RJP attenuates the extracellular Aβ_1-40_ and Aβ_1-42_ accumulation of N2a/APP695 cells. N2a/APP695 cells were treated with 1 μg·mL^−1^, 3 μg·mL^−1^ and 9 μg·mL^−1^ fraction 1 C^I^ RJP for 48 hours, and the extracellular Aβ_1-40_ (**A**) and Aβ_1-42_ (**B**) concentration were detected by an Aβ ELISA assay. Data are expressed as the mean ± SD of three independent experiments. Non-treated N2a/APP695 cells were considered as control. ^**^P < 0.01 and ^***^P < 0.001 vs control. Statistical analyses were performed by a one-way ANOVA test and the addition of Bonferroni multiple comparisons test.

Several RJP fractions were separated from crude RJPs via the HPLC method. We chose the 1 C^I^ fraction as the active ingredient after preliminary functional verification. Our results showed that 1 C^I^ RJP below 40 μg·ml^-1^ did not affect N2a and N2a/APP695 cell viability during 48 hours (Fig. 3). Compared with N2a cells, N2a/APP695 cells produced a large amount of extracellular Aβ_1-40_ and Aβ_1-42_. As shown in Fig. 4, N2a/APP695 cells cultured for 48 hours with fresh medium secreted Aβ_1-40_ (666.53 ± 29.48 pg/mg protein) and Aβ_1-42_ (180.19 ± 9.02 pg/mg protein), as measured through an Aβ ELISA analysis. On the other hand, no exogenous Aβ_1-40_ and Aβ_1-42_ was detected in N2a cell medium after the cells were cultured for 48 hours. At the concentration of 9 μg·ml^-1^, 1 C^I^ RJP reduced the Aβ_1-40_ level from 666.53 ± 29.48 pg/mg to 523.71 ± 31.42 pg/mg protein (P < 0.01, Fig. 4A) and the Aβ_1-42_ level from 180.19 ± 9.02 pg/mg to 120.39 ± 8.60 pg/mg protein (P < 0.001, Fig. 4B), in N2a/App695 cells medium. These results suggest that 1 C^I^ RJP affects Aβ metabolism in N2a/APP695 cells.

### Effects of fraction 1 C^I^ RJP on the mRNA levels of Aβ metabolism related genes

**Figure 5:**
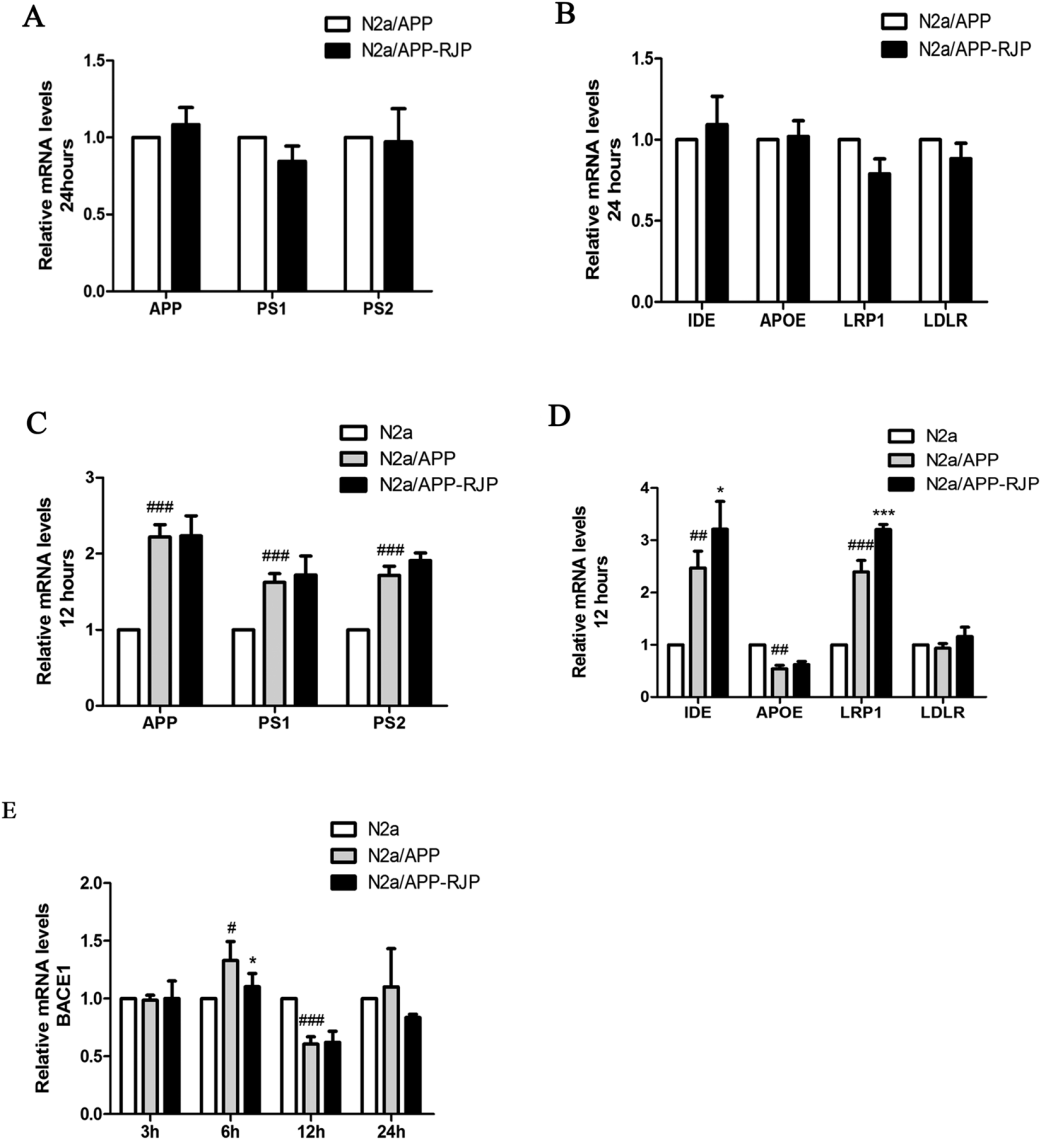
Effects of fraction 1 C^I^ RJP on the mRNA levels of Aβ generation related genes. The relative mRNA expression levels were determined by Real-time PCR. (**A**) Relative mRNA levels of APP, PS1 and PS2, and (**B**) IDE, APOE, LRP1, and LDLR after treatment with 9 μg.mL^−1^ 1 C^1^ RJP for 24 hours. (**C**) Relative mRNA levels of APP, PS1 and PS2, and (**D**) IDE, APOE, LRP1, and LDLR after treatment with 9 μg·mL^−1^ 1 C^1^ RJP for 12 hours. (**E**) Relative mRNA levels of BACE1 after treatment with 9 µg/ml 1 C^1^ RJP for 3, 6, 12, and 24 hours. Non-treated N2a cells were considered as control. Data are expressed as the mean ± SD of three independent experiments. ^*^P < 0.05 and ^**^P < 0.01 vs N2a/APP treated with vehicle, ^#^*P* < 0.05, ^##^*P* < 0.01 and ^###^*P* < 0.001 vs control. Statistical analyses were performed by a one-way ANOVA test and the addition of Bonferroni multiple comparisons test.

In order to investigate how 1 C^I^ RJP down-regulates extracellular Aβ_1-40_ and Aβ_1-42_ accumulation in N2a/APP cells, we performed real-time PCR (qPCR) to determine the change of mRNA levels for Aβ metabolism related genes. We treated the cells with 9 µg/ml 1 C^1^ RJP for 24 or 12 hours after the cells were cultured for 24 hours. Several genes related to Aβ metabolism were examined, including APP, BACE1, Presenilin 1 (PS1), Presenilin 2 (PS2), Insulin-degrading enzyme (IDE), apolipoprotein E (APOE), low density lipoprotein related receptor (LRP1), and low density lipoprotein receptor (LDLR). The mRNA levels of most genes analyzed, including APP, PS1, PS2, IDE, APOE, LRP1 and LDLR, were remain unchanged by the treatment of 1 C^1^ RJP compared with N2a/APP group at 24-hour time point (Fig. 5A,B). However, with a 12-hour 1 C^1^ RJP treatment, gene expression of IDE and LRP1 was significantly up-regulated, whereas expression of APOE was down-regulated; the expression of other genes was still not significantly changed (Fig. 5C,D). Furthermore, we investigated BACE1 expression at 3 hours, 6 hours, 12 hours and 24 hours’ treatment with 9 µg/ml 1C^1^ RJP, results indicated that BACE1 mRNA level was significantly down-regulated after cells were treated for 6 hours (Fig. 5E).

### Fraction 1 C^I^ RJP down-regulates BACE1 protein expression and BACE1 activity

**Figure 6:**
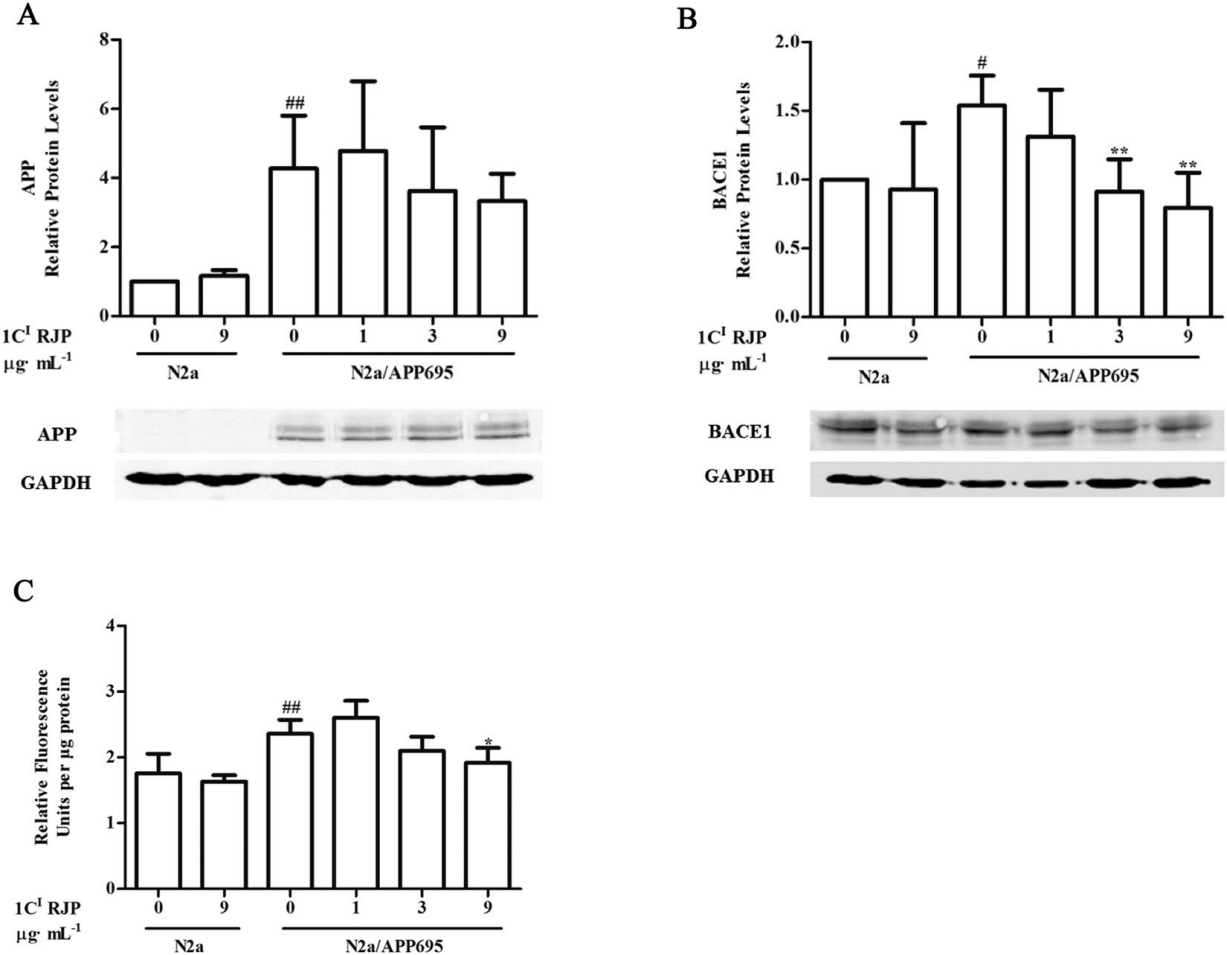
Fraction 1 C^I^ RJP down-regulates BACE1 activity and protein expression on N2a/APP695 cells. Western blot and their corresponding quantifications are shown for APP protein expression (**A**) and BACE1 protein expression (**B**) after N2a/APP695 cells treated with 1 C^I^ RJP fraction for 24 hours. Beta-secretase activity fluorometric assay was performed to detect BACE1 activity (**C**) after N2a/APP695 cells were treated with the 1 C^I^ RJP fraction for 24 hours. Non-treated N2a cells were considered as control. ^#^*P* < 0.05, ^##^*P* < 0.01 vs control, ^*^P < 0.05 and ^**^P < 0.01 vs N2a/APP cells treated with vehicle, Data are expressed as the mean ± SD of three independent experiments. Statistical analyses were performed by a one-way ANOVA test and the addition of Bonferroni multiple comparisons test.

The generation of Aβ depends on the cleavage of amyloid precursor protein (APP) by β-secretase (BACE1) and γ-secretase. For this reason, we thought that RJP may affect BACE1 or γ-secretase expression or their catalytic activities. The γ-secretase complex contains four components: Presenilin 1 (PS1), Presenilin 2 (PEN2), APH-1 and Nicastrin, of which PS1 provides the catalytic function and PEN2 facilitates the autocatalytic cleavage of PS1^28^. We evaluated APP, BACE1, PS1 and PEN2 protein expression by western blot analysis after 24 hours of treatment with 1 C^I^ RJP in both N2a and N2a/APP695a cells. N2a cells and N2a/APP695 cells were regarded as the control group and model group, respectively. Our data showed that APP expression was significantly higher in the model group than in the normal N2a cells (P < 0.01 vs model, Fig. 6A), whereas 1 C^I^ RJP treatment did not change the APP expression in either group. These results indicate that our cell model successfully expressed high levels of APP. Moreover, BACE1 protein expression significantly elevated in N2a/APP695 cells (P < 0.01 vs control, Fig. 6B). When N2a/APP695 cells were treated with 1 C^I^ RJP for 24 hours, the BACE1 level decreased in a dose-dependent manner and was significantly attenuated at 1 C^I^ RJP concentrations of 3 μg·ml^−1^ and 9 μg·ml^−1^ (P < 0.01 vs model, Fig. 6B). However, 1 C^I^ RJP did not affect BACE1 expression in N2a cells (P < 0.01 vs control, Fig. 6B). For PS1 and PSEN2 protein expression, 1 C^I^ RJP did not significantly influence their expression in N2a/APP695 cells (data not shown). We further determined BACE1 activity using a beta-secretase fluorometric assay kit in N2a and N2a/APP695a cells treated or not with 1 C^I^ RJP. Data revealed that BACE1 activity was greatly enhanced in the model cells than control cells, which may be responsible for greater Aβ generation (P < 0.01 vs control, Fig. 6C). Treatment with 1 C^I^ RJP for 24 hours could weaken its activity in model cells but not N2a cells in a dose-dependent manner, and the activity was significantly down-regulated when treated with 9 μg·ml^−1^ of 1 C^I^ RJP (P < 0.05 vs model, Fig. 6C).

### Histone deacetylation is involved in the 1 C^I^ RJP-induced down-regulation of BACE1

**Figure 7:**
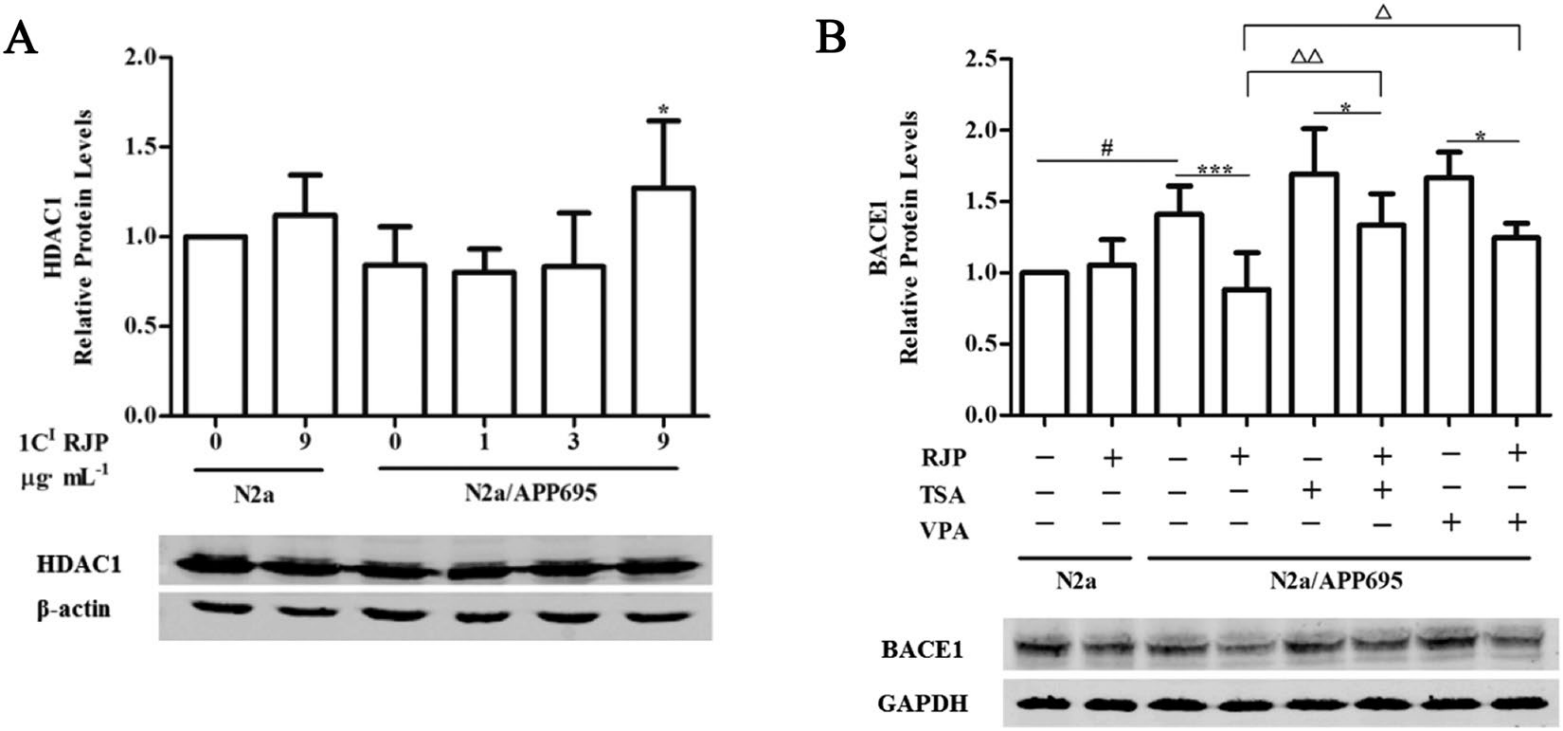
Histone deacetylation is involved in 1 C^I^ RJP-induced down-regulation of BACE1 in N2a/APP695 cells. Western blot and their corresponding quantifications are shown for HDAC1 protein expression (**A**) in N2a or N2a/APP695 cells treated with 1 C^I^ RJP for 24 hours. Also, western blot and their corresponding quantifications are shown for N2a/APP695 cells treated with 1 C^I^ RJP or/and HDAC inhibitors TSA (50 nM) or VPA (1 mM) for 24 hours. HDAC inhibitors were added 30 mins prior to 1 C^I^ RJP addition. Non-treated N2a cells were considered as control. Data are expressed as the mean ± SD of three independent experiments. ^#^*P* < 0.05 vs control; ^*^P < 0.05 vs non-treated N2a/APP695 cells (**A**); ^*^P < 0.05, ^***^P < 0.001, ^Δ^*P* < 0.05 and ^ΔΔ^*P* < 0.01 between the indicated treatments (**B**). Statistical analyses were performed by a one-way ANOVA test and the addition of Bonferroni multiple comparisons test.

The roles of HDACs in cognitive function as well as in neurological disorders and diseases have been demonstrated for years^29^. Considering RJP could down-regulate BACE1 expression, we next determined whether 1 C^I^ RJP is related to histone deacetylation. Thus, we detected HDAC1 and HDAC2 protein expression after treatment of N2a and N2a/APP695 cells with 1 C^I^ RJP for 24 hours. Our data showed that 9 μg/ml 1 C^I^ RJP could significantly enhance HDAC1 protein expression (P < 0.05 vs model, Fig. 7A), whereas 1 C^I^ RJP did not affect HDAC2 expression (data not shown).

To investigate whether HDAC1 plays an important role in 1 C^I^RJP induced down-regulation of BACE1 expression, we designed HDAC inhibitor experiment. TSA and VPA are HDAC inhibitors, which can restrain the down-regulation of BACE1 through boosting histone H3 acetylation^30,31^. We pretreated N2a/APP695 cells with TSA and VPA before 1 C^I^ RJP treatment. We found that the BACE1 protein level increased (P < 0.05 vs control, Fig. 7B) and decreased significantly upon treatment with 1 C^I^ RJP (P < 0.001 vs model, Fig. 7B), which was the same as the above results. Meanwhile, the data indicated that BACE1 expression was higher in the TSA and RJP co-treatment group than in the RJP treatment only group (P < 0.01, Fig. 7B). In the same line, VPA displayed a similar function as TSA (P < 0.05, Fig. 7B). These results suggest that TSA and VPA could strongly prevent the down-regulation of BACE1 by 1 C^I^ RJP treatment. However, BACE1 expression in the TSA or VAP and RJP co-treatment group was significantly lower than in cells only treated with TSA or VAP, which implies that there may be other mechanisms by which RJP regulates BACE1 (P < 0.05, Fig. 7B).

## Discussion

Royal jelly contains several types of proteins, including major royal jelly protein 1 to 9 (MRJP1-9), and MRJP1-MRJP5 account for 82–90% of total royal jelly protein^7^. MRJP1 is the main component of WSRJ protein, with a molecular weight of approximately 55 kDa. Additionally, MRJP2, MRJP3, MRJP4 and MRJP5 are approximately 49 kDa, 60-70 kDa, 60 kDa and 80 kDa, respectively^32^.

Royal jelly is the life-long food of queen bees and first-three-day food for bee larvae. Queen bees can live much longer and have better fertility than normal bees because of eating Royal jelly. It has been demonstrated that major royal jelly protein 1 was the component which plays the main role in bee larvae differentiation^33^. And larvae bees digest and absorbed it by intestinal. As for human being, royal jelly is a kind of health care products. People used royal jelly by oral administration. In order to imitate the process of bee larvae digestion, we used larvae intestinal enzymes complex to enzymolysis royal jelly protein and separate effective peptides.

Royal jelly peptides (RJPs) obtained from royal jelly or major royal jelly protein (MRJP) have been studied in several publications, but few have focused on the neuroprotective effect of RJPs. In this study, we obtained crude RJPs (MW < 1 kDa, 1-3 kDa, and 3-5 kDa) from MRJP digest. Afterwards, 1-3-kDa RJPs were chosen to be purified using an RP-HPLC method, and several RJP fractions were obtained from the original RJPs. Based on preliminary bioactivity selection, we chose fraction 1 C^I^ RJP as the active component to perform the subsequent investigation.

N2a/APP695 cells produced large amounts of Aβ1-40 and Aβ1-42 extracellularly, and no exogenous Aβ1-40 or Aβ1-42 was detected in N2a cell medium after they were cultured for 48 hours. These results were similar to those in a previous study^21^. Unquestionably, 1 C^I^ RJP was purer than crude RJPs after several steps of RP-HPLC purification. Considering the output of 1 C^I^ RJP and its preliminary bioactive concentration, we chose 1, 3, and 9 μg/ml as the suitable concentrations for the subsequent investigation. Our results showed that 1 C^I^ RJP treatment for 48 hours could significantly inhibit the accumulation of extracellular Aβ1-40 and Aβ1-42 of N2a/APP695 cells in a dose-dependent manner. These results suggest that 1 C^I^ RJP affects Aβ metabolism in N2a/APP695 cells. Numerous genetic and biochemical studies have indicated that Aβ overexpression and aggregation can be toxic and have been regarded as one of the key pathological factors of the onset of AD^17^. Thus, RJPs obtained from royal jelly may be beneficial in alleviating the progression of AD.

Multiple gene groups are involved in the complex Aβ metabolism, including its production (APP, PS1, and PS2), degradation (IDE) and transportation (APOE, LRP1, and LDLR). Several key genes were examined in our experiment using real-time PCR. There was no significant change for them in expression in the groups treated with RJP for 24 hours. Perhaps 24-hour time point was not the best for measuring the gene transcriptional levels with RJP treatment, and the transcription process of these genes had already ended. However, when we measured gene expression after 12-hour RJP treatment, the relative gene expression level of IDE and LRP1 were significantly up-regulated. The up-regulation of IDE could accelerate the degradation of Aβ, and the elevation of LRP1 may enhance the efflux of Aβ across the blood brain barrier (BBB) and reduce the Aβ burden in the brain.

Aβ is generated from the serial cleavage of amyloid precursor protein (APP). APP is cleaved by β-secretase (BACE1) and generates the secreted derivative sAPPβ and β-C-terminal fragment (β-CTF). Afterwards, β-CTF is cleaved within the membrane by γ-secretase, leading to the release of Aβ^34^. BACE1 is the key rate-limiting enzyme of Aβ production. The expression level of BACE1 can reflect the production level of Aβ to some extent. The basal transcriptional level of BACE1 in N2a/APP cells after 6 and 12 hours of culturing revealed that BACE1 was highly expressed after 6 hours and inhibited after 12 hours through a cellular stress reaction (Fig. 5E). After treatment with 1C^1^ RJP for 6 hours, mRNA level of BACE1 was down-regulated significantly (Fig. 5E). These results indicated that 1 C^1^ RJP can inhibit BACE1 expression at a specific time point. Interestingly, both a reduction in BACE1 protein expression and enzyme activity induced by 1C^1^ RJP were observed at a later time point as well, which in parallel with inhibition effect of RJP on BACE1 transcriptional level, hence contribute to the reduction of APP cleavage, leading to less Aβ release. The process from transcription to translation may need some time. So the changes of BACE of RNA and protein have shown at different timing. Anyway, all the above results imply that 1C^I^ RJP can inhibit the extracellular Aβ accumulation of N2a/APP695 cells through the down-regulation of BACE1 protein expression and activity. As BACE1 is the key rate-limiting enzyme in the generation of Aβ, blocking BACE1 proteolytic activity will suppress Aβ generation, thereby targeting Aβ pathology for AD therapy. Currently, many potential BACE1 inhibitor drugs have been discovered, such as MK8931, AZD-3293, JNJ-54861911, E2609 and CNP520^35^. Few natural inhibitors have been reported and we take ginsenoside Re for an example, ginsenoside Re in the range of 25-100 μM/l (24 mg/l-95mg/l) can decrease BACE1 protein and mRNA expression in a dose dependent manner^36^. In comparison with it, our RJ peptides can decrease the BACE1 protein in lower concentration despite the peptides are mixed peptides. The single peptide has been analyzed and further experiments are in process now. Considering the bioactivity of 1 C^I^ RJP, it could serve as a new natural BACE1 inhibitor.

Gene expression can be controlled by epigenetic modification. Recent studies have shown that histone acetylation activates BACE1 expression, and there is an increase in histone H3 acetylation in the BACE1 promoter^26,27^. Histone deacetylases (HDACs) can remove acetyl groups from histones, strengthening the binding of DNA with histones, thereby regulating gene expression by inhibiting the binding of DNA and transcription factors^24^. Our data showed that 9 μg/ml 1 C^I^ RJP could significantly enhance HDAC1 protein expression, whereas 1 C^I^ RJP did not affect HDAC2 expression. The HDAC1 inhibitor experiment results suggest that TSA and VPA could strongly prevent the down-regulation of BACE1 induced by 1 C^I^ RJP treatment, but we cannot exclude the possibility that there may be other mechanisms by which RJP regulates BACE1.

Thus, our study found that 1 C^I^ RJP could down-regulate the BACE1 protein level and proteolytic activity to eliminate exogenous Aβ accumulation in N2a/APP695 cells. Additionally, 1 C^I^ RJP may affect BACE1 expression through a histone deacetylation modification mechanism. However, the exact mechanism by which RJP regulates BACE1 expression should be investigated further. Even though we verified that the relatively pure RJP functioned well in the present study, we haven’t elucidated its sequence and structure information. Obviously, we should explore how to obtain monomer peptides that act on BACE1. In addition, animal model experiments should also be considered to test RJP activity in future study. In summary, all the above results suggest that RJPs are neuroprotective, and royal jelly could be a source of RJPs, which could be regarded as a potential natural product to relieve neurodegenerative diseases such as AD in the elderly.

## Materials and Methods

### Chemicals and materials

Ultrapure water was produced by a Milli-Q system (Millipore, USA). HPLC-grade acetonitrile (ACN) (Fisher, USA) and trifluoroacetic acid (TFA) (Sigma, USA) were used. The BCA kit and western blot gel buffers were purchased from Beijing CellChip Biotechnology Company (Beijing, China). Cell culture plates were purchased from Nest Biotechnology Company (Jiangsu, China). All other reagents and chemicals (G418) were purchased from Sigma Chemicals Company (Sigma, USA).

### Preparation of the *Apis mellifera* larva intestinal enzyme

The procedure was performed as following steps: Briefly, 2-3 days old *Apis mellifera* larvae were washed with cold 0.9% normal saline, and the intestinal canal was isolated from the larvae (Mentougou district, Beijing, China). The intestinal canal was mixed with 50 mM PBS (pH = 7.0) at a ratio of 1: 1, and was ground using an electric homogenizer in an ice bath. Then, the mixture was centrifuged at 20000 *g* for 20 min at 4 °C, and the middle layer solution was collected. Centrifuge the middle layer solution again in the same conditions, and a yellow transparent liquid was obtained, which was the intestinal enzyme solution. The mixture was stored at −20 °C.

### Preparation of the water-soluble royal jelly protein

Royal jelly and 50 mM pH 7 PBS solution (1:2, v/v) were mixed, and the mixture was centrifuged at 20000 *g* at 4 °C for 20 min to remove the insoluble ingredients. The supernatant was dialyzed with a dialysis bag with a molecular weight cut-off of 8000 to 14000 Da (MD77 MM, Millipore, USA) in an ice bath with 50 mM pH 7 PBS for 48 hours. The PBS solution was replaced every 12 hours. The solution in the dialysis bag was stored at −20 °C.

### Digestion of the water-soluble royal jelly protein

Several kinds of proportion of water soluble royal jelly protein and intestinal enzyme mixture had been tried as following: enzyme 15 mg and WSRJ 20 mg(lane B), enzyme 15 mg and WSRJ 40 mg(lane C), enzyme 15 mg and WSRJ 60 mg (lane D), enzyme 15 mg and WSRJ 80 mg(lane F).The WSRJ was about 42.7 mg/ml while intestinal enzyme mixture was about 31.9 mg/ml. The total volume was 10 ml, except for those two components, we added PBS to 10 ml. The optimized digestion condition for the intestinal enzyme solution and water-soluble royal jelly protein (1:3 mg/mg) mixture was 37 °C, pH 8.5 for 24 hours in a 2000-mL container. The reaction was stopped by placing the mixture in an ice bath, and the crude royal jelly peptides were obtained. This solution was centrifuged at 10000 *g* at 4 °C for 10 min to remove the insoluble components. Additionally, a 10 μm (Millipore, USA) microfiltration membrane was used to eliminate impure contents. Afterwards, crude RJPs were preliminarily purified through a 5-kDa, 3-kDa and 1-kDa ultra-filtration column and RJPs with MW < 1 kDa, 1-3 kDa, and 3-5 kDa were obtained (MSM-2008, Shanghai Mosu Science Equipment, China). Then, the digests were lyophilized and reconstituted in distilled water. In addition, the digests were filtered using a 0.22 μm (Millipore, USA) membrane, and the protein concentration was measured using a BCA assay.

### Crude peptides were purified using the RP-HPLC method

The obtained 1-3 kDa RJP was purified using the HPLC system (LC2000, CXTH, Beijing, China) with different sizes of chromatography columns. The mobile phases were as follows: (A) deionized water with 0.5% TFA and (B) acetonitrile with 0.5% TFA. The HPLC system consisted of two pumps and a UV detector. We used a 220-nm detection wave length in all the purification processes.

For the first separation, a 35 × 50 mm low-pressure glass column was employed with the C18 material, 20 μm, 300 Å (ODS-A, YMC, Japan). The elution condition was 100% A for 30 min, 25% B for 20 min and 55% B for 20 min at a flow rate of 20 mL/min.

Then, a 250 × 20 mm, S-10 μm, 12 nm C18 column (ODS-A, YMC, Japan) was used for the next stage of preparation, and the mobile phase, 10% B for 16 min and 60% B for 20 min at a flow rate of 10 mL/min, was applied for the purification process.

Afterwards, semi-RPLC was performed to obtain a more purified RJP using the 250 × 10 mm, S-5 μm, 12 nm C18 column (ODS-A, YMC, Japan). We used a gradient elution procedure: 30-80% B for 15 min with a flow rate of 3 mL/min.

Finally, analysis was carried out with the following gradient: 5–60% B for 60 min at a flow rate of 0.5 mL/min using a 250 × 4.6 mm, S-5 μm, 12 nm C18 column (CXTH, China).

### Cell culture and treatment

Mouse neuroblastoma N2a cells and N2a cells stably expressing human APP genes (N2a/APP695) were donated by Professor Zhang Wensheng’s laboratory (Beijing Normal University, China). N2a cells were cultured in Opti-MEM and DMEM-H medium (1:1, v/v, Gibco, USA) supplemented with 5% (v/v) fetal bovine serum (PAA, USA), 100 U/mL penicillin, 100 μg/mL streptomycin and 1% GlutaMAX (Gibco, USA) at 37 °C in a humidified atmosphere containing 5% CO_2_. N2a/APP695 cells were cultured in the same conditions except with 500 μg/mL G418 (Amresco, USA). The cells were passaged every 2-3 days when cells reach 80% confluence. Before drug treatment, cells were plated and allowed to grow for 24 hours. Then, cells were treated with RJPs for 24 hours or 48 hours with fresh medium. In addition, cells were co-treated with RJPs and histone deacetylase inhibitors (trichostatin A, TSA, 50 nmol•L^−1^ or valproic acid, VPA, 1 mmol•L^−1^) for 24 hours after the cells were pretreated with these inhibitors (TSA, Sigma, USA; VPA, Selleck, USA) for 30 min.

### Cell viability assay

Cells (3 × 10^4^ cells/ml) were plated in 96-well plates and allowed to grow for 24 hours. Cells were treated with different RJP fractions at a suitable concentration for 24 hours or 48 hours. Cell viability was then assessed using the MTT (3-(4,5-Dimethylthiazol-2-yl)-2,5-Diphenyltetrazolium Bromide) assay. Here, 5 mg/mL MTT (Amresco, USA) was added, and the cells were incubated for 4 hours at 37 °C. Afterwards, 150 μL DMSO was added to each well, and the optical density (OD) was measured using a microplate reader (Thermo Labsystems Multiskan MK3, USA) with a 630-nm filter. Cell viability was calculated as relative cell viability = (OD treated − OD blank) × 100%/(OD control − OD blank).

### Aβ ELISA assay

N2a/APP695 cells (5 × 10^4^ cells/mL) were cultured in a 12-well plate. Conditioned media from RJP-treated and -untreated N2a/APP695 cells for 48 hours were collected. Then, the concentrations of Aβ1-40 and Aβ1-42 were quantified using human Aβ ELISA kits (Invitrogen, USA)^22^. The optical density at 450 nm of each well was obtained on a plate reader (FLx800, BioTeK, USA). The concentrations of Aβ1-40 and Aβ1-42 were calculated by comparing with the Aβ1-40 and Aβ1-42 standard curves. All OD values were in the linear range of the assay. The concentrations of Aβ1-40 and Aβ1-42 are shown as pg/mL protein.

### Beta-secretase activity fluorometric assay

N2a/APP695 cells (1.35 × 105 cells/mL) were cultured in 60-mm dishes for 24 hours and were then treated with RJPs for 24 hours. Cells were collected to evaluate BACE1 activity according to the Beta-Secretase Activity Fluorometric Assay kit (Biovision, USA). BACE1 activity was expressed as the relative fluorescence units per μg of protein sample.

### Real-time PCR

RNA was extracted using a TRIzol Reagent RNA Extraction Kit (TIAN GEN), and total RNA (100 ng/μL) was converted to cDNA using the Fast Quant cDNA Reverse Transcription Kit (TIAN GEN). Amplification was performed using the Fast 7500 Real-Time PCR System (ABI, USA) and analyzed using the ΔΔC_T_ method. The primers were designed using Primer 5.0 software and synthesized by BGI (Beijing) and were as Tables 1–3.

**Table 1:** Primer sequences.

Gene	Primer sequence
GAPDH	F: 5′ GGTTGTCTCCTGCGACTTCA 3′
	R: 5′ TGGTCCAGGGTTTCTTACTCC 3′
APP	F: 5′ CCTGATAAACTTCCCACGACA 3′
	R: 5′ CTCTGCCTCTTCCCATTCTCT 3′
BACE1	F: 5′ ACCTATGCGATGCGAATGTT 3′
	R: 5′ AGATGGGCTTCTGTCTTGGAG 3′
PS1	F: 5′ TTCTACTTCGCCACGGATTAC 3′
	R: 5′ GGGCTTGCTCTCTGTTTTTGT 3′
PS2	F: 5′ CACTATCAAGTCTGTGCGTTTCTA 3′
	R: 5′ GGTGTTAAGCACGGAGTTGAG 3′
IDE	F: 5′ AGTTCCCTGAGCACCCTTTC 3′
	R: 5′ GGTATTCACCCAGCCCTTTG 3′
APOE	F: 5′ AACCGCTTCTGGGATTACCT 3′
	R: 5′ CATCAGTGCCGTCAGTTCTTG 3′
LRP1	F: 5′ AAAAGGGCATTTATCAACGG 3′
	R: 5′ CCAAAACAGATTTCGGGAGA 3′
LDLR	F: 5′ CAGGCTCCAAGCATCCATT 3′
	R: 5′ GCCAGAAACCACCAAACAAA 3′

**Table 2:** Reaction mixture:

Reaction Component	Concentration	Volume (µL)
SybrGreen qPCR Master Mix	2X	10
Primer F (10 µM)	10 µM	0.4
Primer R (10 µM)	10 µM	0.4
ddH20		7.2
Template (cDNA)		2

Total 20 µL.

**Table 3:** Reaction protocol.

Thermal Cycler	Time and Temperatures			
	Initial Steps	Each with 40 cycles		
		Melt	Anneal	Extend
ABI StepOne plus Real-time PCR system	Hold	Cycle		
	3 min 95 °C	7 sec. 95 °C	10 sec. 57 °C	15 sec. 72 °C

### Western blot analysis

N2a/APP695 cells (1 × 10^5^ cells/mL) were cultured in a 6-well plate for 24 hours and were then treated with RJPs for 24 hours. Cells were washed with cold PBS and lysed with 1 × loading buffer (10 mM Tris, pH 6.8; 1%SDS; 5% glycerin; 0.1 M DTT; bromophenol blue; 1 mM AEBSF (Sigma, USA)). The lysate was collected and boiled at 100 °C for 10 min. Subsequently, an equal volume of sample was electrophoretically separated using 10% SDS-PAGE and then transferred to an NC membrane (Millipore, USA). Membranes were incubated overnight at 4 °C with various primary antibodies. The primary antibodies used in this study: mouseanti-APP (6E10, 1:250, Covance, USA), rabbit-anti-BACE1 (1:500, Abcam, USA), rabbit-anti-HDAC1 (1:5000, AB clonal, Wuhan, China), rabbit-anti-β-actin and rabbit-anti-GAPDH (1:2000, Cell Signaling Technology, USA). The secondary antibodies used were goat-anti-mouse IgG IRDye 800CW (1:10000, Li-COR, USA) and goat-anti-rabbit IgG IRDye 800CW (1:10000, Li-COR, USA). After incubation with the secondary antibody, the membranes were washed 4 times for 5 min each time. Finally, we used the LI-COR Odyssey Infrared Fluorescence Scanning System (Li-COR, USA) to quantify the fluorescence intensity. The intensity of the bands was analyzed using the system software.

### Statistical analysis

All assays were repeated at least three times, and all values are represented as the mean ± SD. Statistical analysis was performed using a one-way analysis of variance (ANOVA), and multiple comparisons were made using the Bonferroni’s test. A P value less than 0.05 was considered statistically significant. All statistical analyses were performed using SPSS 20.
